# Mr. Munsford on the Production of Animal Heat

**Published:** 1813-06

**Authors:** J. G. Munsford

**Affiliations:** Frome


THE
### Medical and Physical Journal.

6 OF VOL. XXIX.] JUNE, 1813. [NO. 172.

*To the Editors of the Medical and Physical Journal.*

GENTLEMEN,

THE following observations were occasioned by a paper which appeared in your Number for February, on the influence of the brain on the generation of animal heat, by Mr. Brodie, which, if you think worthy a place in your Journal, I shall feel obliged by your inserting.

Mr. B., in a series of well-conducted experiments, has ascertained with great precision the relative quantities of air consumed by animals in a natural state, and when the brain has ceased to perform its office, either by having its communication with the body completely cut off, or its functions only "disturbed" by the effects of poison. He has also ascertained, that in the two latter cases, though the circulation goes on, and the usual chemical changes continue to take place in the blood, no heat is generated. The argument drawn from this is, that the brain has a greater share in the production of animal heat than has hitherto been suspected.

Before I proceed farther, I may just take notice, that the effect of such a poison as described by Mr. B. on the brain, must not be looked upon as a mere "disturbance" of its functions, but a direct attack on its vitality. This is an important distinction. The functions of the brain may be disturbed to a very great degree, as I shall have occasion to notice, without affecting the temperature of the body; but the effect of the poison of woorara, or the essential oil of almonds, is to produce an abolition of all the functions of the brain, a dissolution of the powers of life, whether existing in the brain or elsewhere; and which admit of being restored or not, by supporting an artificial respiration, as the effect of the poison has been more or less powerful. This experiment with the poison then, is the same as the first, where the brain is separated from the body: in both cases the subject of experiment is no longer a living body: we must look for the action of the heart, and the changes which for a certain time continue to take place in the two circulations, amongst other causes, and may not find it any matter of surprise that the power of generating heat is extinct.

When the communication between the head of an animal and its body is interrupted, the heart presently ceases to act, and and why? Not at that instant, because it is deprived of the influence of the brain; for the heart possesses in itself a principle of action independant of the brain, and this for a certain time even out of the body; but because it has lost its natural stimulus, the influx of oxydised blood. The influence of the brain necessary to the action of the muscles of the thorax is lost; respiration consequently ceases; no more blood is propelled towards the left side of the heart, which is now at rest. But prevent this from taking place, support an artificial respiration, force but a little blood from the lungs into the heart, and the heart pursues it from auricle to ventricle, and through the circulation, as long as its contractile power exists. But we must not lose sight of the true nature of this circulation. The body, under such circumstances, is to be considered as little better than an hydraulic machine, the vessels approaching to the state of dead tubes, and the heart, like the syringe of the anatomist, giving motion to the whole. Yet the heart is not wholly unassisted in this office; the arteries too, retaining for a short time a portion of their original irritability and contractile power, become obedient to their wonted stimulus, and feebly contracting upon the volume of oxydised blood, assist in pushing it onwards, to complete the circulation.

But that this kind of circulation would produce heat, is more surely than any one now would venture to expect; and when it is found by experiment not to do so, there is no occasion for supposing that the brain and nerves are the only agents in this manufacture of heat, and that the blood in the living state is no party concerned. This Mr. B. does not do; on the contrary, he pronounces such a conclusion absurd. But it appears to me to be what a slender physiologist would be very apt to do from these experiments, or otherwise be at a loss what he was to infer.

A knowledge of the fact that the usual chemical changes take place in the blood independent of the brain, furthers us but a very little in the physiology of animal heat. To prove it independent of the one, is by no means to establish it as the product of the other. All the changes purely chemical which happen in the blood, take place as well out of the body as within it, till putrefaction commences. The consumption of oxygen, the giving off of carbonic acid, and the consequent changes in appearance resembling that from venous to arterial blood, and *vice versâ*, are the simple products of chemical affinity, unaccompanied by any evolution of heat. It would be folly, then, to expect that heat should be produced in the living body by the same simple process. But that heat does nevertheless depend essentially upon upon respiration, and the circulation of the blood, cannot admit of a doubt; and not only does it essentially depend upon these two, but if the various arguments in favor of their agency, and those which favor that of the brain, be fairly brought into view, I think they will be found to bear much in favor of the former. I shall endeavour to sketch a few which present themselves in this cursory view of the subject.

It is a law in nature, to which I believe there is no exception, that the temperature of every kind of animal is proportioned to the quantity of air which it breathes in a given time. Fishes, who breathe but very little, have their temperature exceedingly low, but little raised above the medium in which they live. Man, and the larger animals, who breathe a great quantity of air, have a high temperature, and differing but little one from another. Birds, again, though living in the same medium, have their temperature five or six degrees higher; and this, as it would seem, not to answer any particular purpose in their economy different from other animals, but as the necessary consequence of their peculiar mode of respiration; of the large quantity of air which they breathe, assisted in its action upon the blood by pressure in its passage to and from the air-cells, all the air in filling and emptying of which must pass with great rapidity through the lungs, and of course with more effect than in the ordinary mode of respiration.

Now, since it is proved that the temperature of all animals is in an exact ratio with the quantity of air which they breathe, and the powers employed in this function, it would be but a fair analogy to conclude, that if this power of generating heat were to be transferred to the brain, we might expect to find that it too would hold some relative proportion to the size and temperature of the animal which it had to supply. But this is by no means the case. It may soon be perceived by any one but a little acquainted with comparative anatomy, that this variety of temperature happens without any relation to the different conditions of the brain. The long list of quadrupeds, with every variety of structure, and relative proportion of brain, differ but little in temperature one from another. Man, whose brain is so much larger in proportion to his size than that of all other animals, is not warmer than those last mentioned; and birds, whose temperature is higher than all, have their brain the least in proportion to their size of any. The inference from all this seems plain, viz. that the brain can have no exclusive or predominant influence in the production of animal heat.

This argument appears to receive additional strength from

 the the phenomena of certain diseases. Here too we might expect to find, that as the brain was either free and undisturbed in its operations, or oppressed and disordered, the heat of the body would fluctuate. But neither is this necessarily the case. Where the brain is in the most depressed state it can be in consistent with life, its functions partly destroyed, and its energy and influence of course greatly diminished, as in apoplexy; while the force of the circulation continues, the heat of the body remains unaltered. In hemiplegia, it is no unusual thing to find the paralytic limb of one side, of the same temperature as the sound one of the other; and, on the other hand, where the brain is in the greatest possible state of excitement, as in phrenitis, where its powers and influence on the body are so increased that the strength of an otherwise weak person becomes absolutely Herculean, the heat of the body is only altered, as it is in other violent inflammations.

But if there be any error in the circulation of the blood, any cause, as in malconformation of the heart or lungs, by which the blood is sent into the circulation poorly oxydised, the first symptoms which strike us at sight of the miserable subject of such a disease, are the blue color of the skin, and a coldness which nothing can overcome: a coldness not dependant on the casual feelings of the patient, not relative or confined to the surface, but a positive reduction of temperature throughout the substance of the body.

The arguments which have been here advanced, and many more might be added, seem to place the doctrine of animal heat where it stood before, as dependant principally upon respiration and the circulation of the blood; but in what manner is yet perfectly inexplicable, and perhaps may for ever lay beyond the reach of human research. All inquiries hitherto made into this most difficult of all subjects, have but plunged us still deeper into perplexities.

It does not appear then, that the generation of animal heat exists in the brain and nerves as a distinct faculty. It is not in respiration singly; nor in the different capacities for heat of the blood, in its venous and arterial states, as Mr. B.'s experiments sufficiently prove. Neither is it in the constant changes going on in the system in the process of nutrition; the conversion of fluids into solids, and the continual assumption of new parts: for as fast as new parts are added, old ones must be removed; and the caloric disengaged in the conversion of a fluid into a solid to form a new part, must again be taken up, and become latent in the old one, as it changes its state from a solid to a fluid, to be carried out of the system.

I have

I have once or twice, in the course of these observations, spoken of the oxydisement of the blood; a doctrine which, though some late experiments seem to render untenable, we must not be in haste to relinquish.

If the quantity of carbonic acid given out in respiration, equals volume for volume the quantity of oxygen gas consumed, (a cubic inch of one requiring exactly a cubic inch of the other for its formation,) I say, if this be true, and it is to be inferred from it that no oxygen enters into combination with the blood—whence does the fœtus derive its oxygen?—for it would be idle to talk of its living without it.

I shall not trespass farther on your valuable pages, but conclude these remarks with the hope that an unwearied investigation, and the united labors of individuals, may at length throw a little light on this obscure part of physiology. We are already much indebted to Mr. Brodie, whose diligent inquiries, and accuracy of experiment, I hope may entitle us to expect more. I am, Gentlemen, with much respect,

Your obedient humble Servant,

*Frome, April* 13, 1813. J. G. MUNSFORD.

